# Non-invasive cell type selective *in vivo* monitoring of insulin resistance dynamics

**DOI:** 10.1038/srep21448

**Published:** 2016-02-22

**Authors:** Meike Paschen, Tilo Moede, Barbara Leibiger, Stefan Jacob, Galyna Bryzgalova, Ingo B. Leibiger, Per-Olof Berggren

**Affiliations:** 1 The Rolf Luft Research Center for Diabetes and Endocrinology, Karolinska Institutet, SE-171 76 Stockholm, Sweden

## Abstract

Insulin resistance contributes to the development of cardio-vascular disease and diabetes. An important but unresolved task is to study the dynamics of insulin resistance in selective cell types of insulin target tissues *in vivo*. Here we present a novel technique to monitor insulin resistance dynamics non-invasively and longitudinally *in vivo* in a cell type-specific manner, exemplified by the pancreatic β-cell situated within the micro-organ the islet of Langerhans. We utilize the anterior chamber of the eye (ACE) as a transplantation site and the cornea as a natural body-window to study the development and reversibility of insulin resistance. Engrafted islets in the ACE that express a FoxO1-GFP-based biosensor in their β-cells, report on insulin resistance measured by fluorescence microscopy at single-cell resolution in the living mouse. This technique allows monitoring of cell type specific insulin sensitivity/resistance in real-time in the context of whole body insulin resistance during progression and intervention of disease.

Insulin resistance is a key-contributing factor to a variety of metabolic diseases including cardio-vascular disease and diabetes mellitus type 2 (T2DM). A major challenge in the field of insulin resistance is to monitor this process dynamically in individual cell types of insulin target tissues that are composed of different cell types, such as fat, liver, brain, kidney or pancreatic islets in the living organism. Research by us and others has shown that the multicellular micro-organ the pancreatic islet of Langerhans itself is a target for insulin action. Here insulin as an autocrine factor directly affects the β-cell at the level of gene expression, ion flux, glucose metabolism, proliferation, survival and insulin secretion (reviewed in^1^). Moreover, insulin as a paracrine factor affects glucagon release from α-cells^2^ and is discussed to cause vasodilation of islet blood vessels by affecting islet endothelial cells^3^. Consequently, factors leading to islet/β-cell insulin resistance will cause/contribute to β-cell dysfunction and finally to the development of T2DM.

The aim of the present manuscript was to establish and validate a technique to monitor the dynamics of insulin resistance in the pancreatic β-cell *in vivo* in the context of overall body insulin resistance and glucose homeostasis during the progression of T2DM in real-time. We took the advantage of our recently developed imaging platform that allows reporting on pancreatic islet/β-cell function and survival non-invasively, longitudinally and at single-cell resolution *in vivo*^4^,^5^. We transplant pancreatic islets into the anterior chamber of the eye (ACE) of mice for functional microscopic imaging. When pancreatic islets are transplanted into the ACE, β-cell function and survival can be readily imaged and the engrafted islets serve as representative reporters of *in situ* islets in the pancreas^4^,^5^,^6^,^7^,^8^,^9^,^10^. In the present study, we transduced pancreatic islets with adenoviruses that encode a FoxO1-GFP-based biosensor^11^,^12^,^13^ prior to their transplantation into the ACE to monitor insulin resistance in pancreatic β-cells in the living animal. This technique combined with overall insulin tolerance tests allows to monitor the dynamics of β-cell insulin resistance in the context of overall insulin resistance in the development and progression of T2DM.

## Results

### The Pancreatic β-cell Insulin Resistance Biosensor (βIRB)

**Figure 1:**
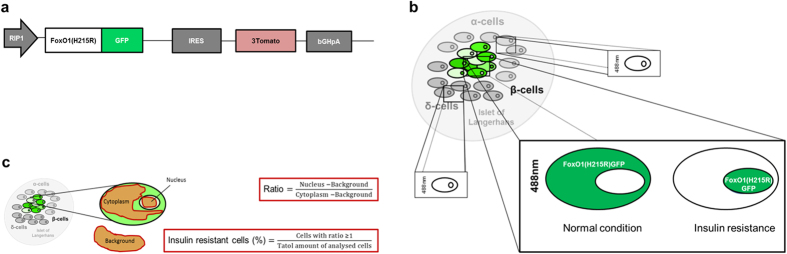
Cell-type selective analysis of insulin resistance in the islet of Langerhans. (**a**) Schematic illustration of β-cell insulin resistance biosensor (βIRB). β-cell specific expression is ensured by the rat-insulin 1 promoter (RIP1). FoxO1 was mutated to FoxO1(H215R) to avoid DNA-binding and coupled to GFP for its detection. 3Tomato was introduced as reference signal for identification of transduced cells. An IRES-element ensures stoichiometric expression of the two fluorescent indicators under the same promoter. (**b**) MIN6 cells and pancreatic islets are transduced with βIRB-encoding adenoviruses. Upon excitation with 488 nm, the fluorescent indicator GFP is identified by laser-scanning fluorescent confocal microscopy. FoxO1(H215R)GFP is cytoplasmic under normal conditions and shuttles into the nucleus at insulin resistance. c) For the analysis of *in vitro* and *in vivo* imaging data, three regions of interest (nucleus, cytoplasm and background) were placed for every cell. The obtained intensities were used for calculation of a ratio. The ratios were categorized into two groups: 1) Ratios < 1, where less FoxO1(H215R)GFP is present in the nucleus than in the cytoplasm (=insulin sensitive) and 2) Ratios ≥ 1, where equal/more FoxO1(H215R)GFP is present in the nucleus compared to the cytoplasm ( = insulin resistant). The percentage of insulin resistant cells was calculated for each animal and for each experimental condition.

In order to monitor insulin resistance in the pancreatic β-cell *in vivo* we took advantage of the features of the ACE model that allows to study β-cell function by fluorescence microscopy non-invasively, longitudinally and at single-cell resolution. Consequently, we aimed for a genetically encoded fluorescent biosensor that allows differentiation of β-cells that are insulin responsive from those that are insulin resistant. A common denominator for insulin resistance at the cellular level is decreased activation of Akt by insulin. One of the important targets of insulin-stimulated Akt is FoxO1^11^,^12^. Phosphorylation of FoxO1 by Akt leads to nuclear exclusion and cytosolic sequestration^13^. Based on the different intracellular localization of FoxO1 in response to insulin, i.e. cytosolic in insulin responsive cells and nuclear in insulin resistant cells, which can be resolved by our ACE *in vivo* imaging platform, we generated a β-cell specific biosensor for insulin resistance (βIRB). This biosensor consists of a mutated form of FoxO1 that does not allow binding as a transcription factor to FoxO1-target genes, i.e. FoxO1(H215R)^14^, which is fused with GFP. As an additional reference signal we introduced the red fluorescent protein Tomato. The cDNAs of FoxO1(H215R)GFP and Tomato were linked by an IRES-sequence thus ensuring stoichiometric expression of both proteins in the same cell. β-cell specific expression of βIRB was ensured by the use of the rat insulin-1 gene promoter (−410/+1 bp) in the adenovirus vector. A schematic illustration of the biosensor and the imaging approach is shown in Fig. 1a,b.

### *In vitro* Assessment of βIRB in MIN6 Cells

**Figure 2:**
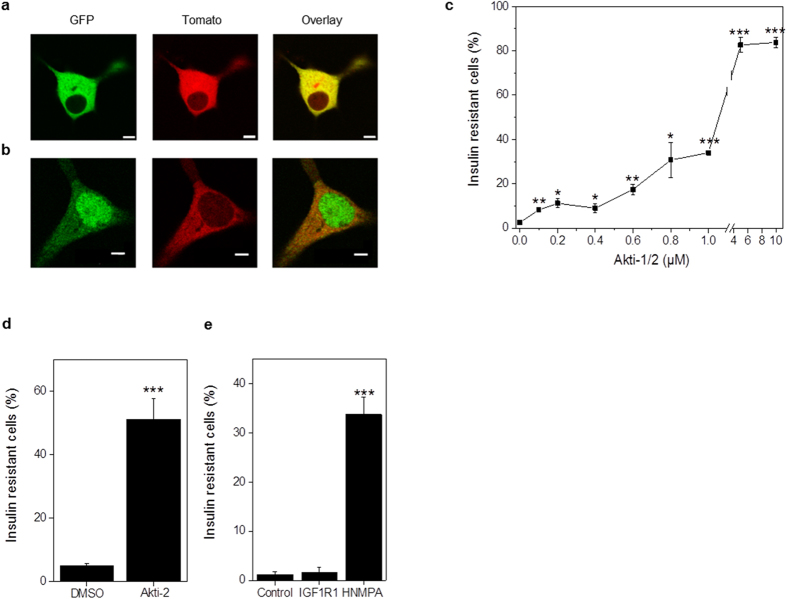
*In vitro* characterization of βIRB in MIN6 cells. Transduced MIN6 cells under (**a**) normal culture conditions or (**b**) after treatment with the Akt-inhibitor (n = 50). (**c**) β-cell insulin resistance in transduced MIN6 cells after Akti-1/2 treatment in a dose-dependent matter (n = 3 experiments). (**d**) β-cell insulin resistance in transduced MIN6 cells treated with the Akt2 inhibitor Akti-2 (n = 3 experiments). (**e**) β-cell insulin resistance in transduced MIN6 cells treated with IGF-1R inhibitor (IGF1R1) and insulin receptor tyrosine kinase inhibitor HNMPA(AM)_3_ (HNMPA) (n = 3 experiments). Data are shown as mean ± SEM, *p < 0.05, **p < 0.01, ***p < 0.001. Scale bars, 5 μm.

In order to test the function of the biosensor *in vitro*, we expressed βIRB in the mouse insulinoma cell line MIN6. These cells express insulin receptors and IGF-1 receptors. They are responsive in terms of insulin signaling by activating among other targets Akt and phosphorylation of its substrate FoxO1^15^. MIN6 cells were transduced with an adenovirus encoding the biosensor. Images were obtained by confocal microscopy using a 63 × 1.2 NA objective. Green fluorescent signals were analyzed in individual focal planes as the ratio of nuclear versus cytosolic intensities. Cells were grouped into insulin responsive (ratio < 1) versus insulin resistant (ratio ≥ 1). The percentage of insulin resistant β-cells was calculated from the ratio of insulin resistant cells to all cells analyzed (Fig. 1c). As shown in Fig. 2, insulin responsive MIN6 cells show a cytosolic localization of both FoxO1(H215R)GFP (green signal) and the reference Tomato (red signal). Decreasing Akt activity by incubating MIN6 cells with the pharmacological inhibitor Akti-1/2 (10 μM) and thus mimicking insulin resistance led to an increase in the nuclear localization of FoxO1(H215R)GFP (green signal), while the reference Tomato (red signal) remains cytosolic (Fig. 2a,b). Since MIN6 cells express all three isoforms of Akt, i.e. Akt1-3, and all Akt isoforms are activated by insulin^16^, we next tested which Akt isoform(s) is responsible for the regulation of βIRB. The pharmacological inhibitor Akti-1/2 selectively blocks individual Akt isoforms at different concentrations, i.e. IC_50_ = 58 nM, 210 nM, and 2.12 μM for Akt1, Akt2 and Akt3, respectively. As shown in Fig. 2c, incubation of MIN6 cells with 0.1 μM Akti-1/2, which inhibits Akt1, already led to a small but significant increase in nuclear localization of the biosensor thus reflecting an increase in insulin resistant cells. Increasing the concentration of the inhibitor to 0.8 μM, thus inhibiting both Akt1 and Akt2, increased number of insulin resistant cells to 40%. Finally, inhibiting all three Akt isoforms by using 5 and 10 μM Akti-1/2 resulted in an increase of nuclear FoxO1(H215R)GFP, i.e. 80% insulin resistant β-cells. This implied a role of all three Akt isoforms in the regulation of the biosensor in MIN6 cells. As shown in Fig. 2d, incubation of MIN6 cells with 4 μM Akti-2, which selectively inhibits Akt2, led to an increase in insulin resistance in 50% of the analyzed cells. To test the involvement of signal transduction via insulin receptors or IGF-1 receptors in the regulation of βIRB, we incubated MIN6 cells with the insulin receptor inhibitor HNMPA(AM)_3_ (100 μM, 1 h) or with the IGF-1 receptor inhibitor PPP (2.4 μM, 1 h). As shown in Fig. 2e, blocking signal transduction through insulin receptors led to an increase in nuclear FoxO1(H215R)GFP, i.e. an increase in insulin resistance in 35% of the analyzed cells, while blocking signalling via IGF1 receptors did not change the cytosolic localization of the biosensor.

### *In vitro* Assessment of βIRB in Isolated Mouse and Human Pancreatic Islets

**Figure 3:**
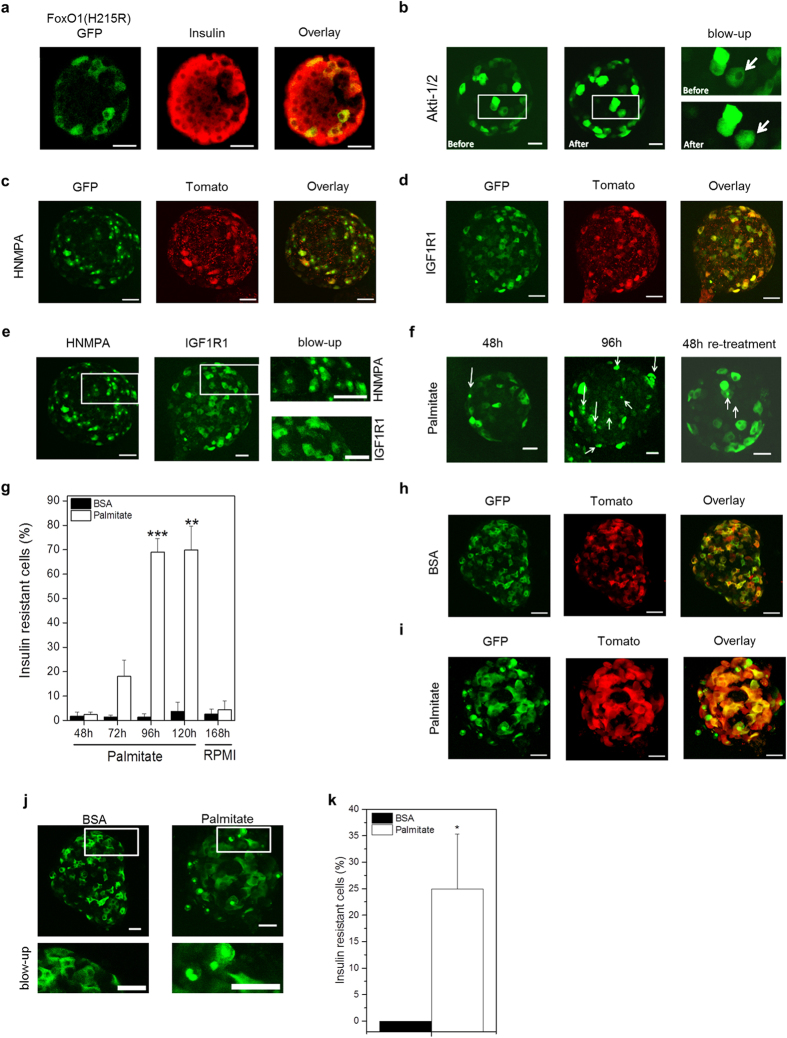
*In vitro* characterization of βIRB in pancreatic islets. (**a**) Representative single focal plane of an isolated islet, transduced with βIRB-encoding adenovirus and immuno-stained for insulin (n = 10). (**b**) Representative maximum projection of an isolated mouse islet transduced with βIRB imaged before and after incubation with Akti-1/2 (n = 3). Change in nuclear localization of FoxO1(H215R)GFP is shown in blow-ups and indicated by an arrow. (**c**) Representative maximum projection of an isolated mouse islet transduced with βIRB and imaged after treatment with HNMPA(AM)_3_. (n = 3) (**d**) Representative maximum projection of an isolated mouse islet transduced with βIRB and imaged after treatment with IGF-1R inhibitor (IGF1R1) (n = 3). (**e**) Representative maximum projection of isolated mouse islets transduced with βIRB and imaged after treatment with HNMPA(AM)_3_ or IGF-1R inhibitor (IGF1R1). (**f**) Representative maximum projection of mouse islets transduced with the biosensor and treated with palmitate for 48 h and 96 h and 48 h after transfer to RPMI after treatment (re-treatment). Nuclear FoxO1(H215R)GFP-positive cells are indicated by arrows (n = 4). (**g**) Effect of palmitate treatment and medium re-treatment on subcellular distribution of FoxO1(H215R)GFP (n = 3). (**h**) Representative single focal planes of human islets transduced with the biosensor under normal culture conditions (n = 5). (**i**) Representative single focal planes of human islets transduced with the biosensor after treatment with palmitate (n = 5). (**j**) Representative single focal planes of human islets transduced with the biosensor under normal culture conditions or after treatment with palmitate (n = 5). (**k**) Effect of palmitate treatment for 144 h on subcellular distribution of FoxO1(H215R)GFP in human islets (n = 5). Data are shown as mean ± SEM, *p < 0.05, **p < 0.01, ***p < 0.001. Scale bars, 30 μm.

We next studied the function of the biosensor *in vitro* in isolated pancreatic islets. Islets were transduced with the biosensor encoding adenovirus. Images were obtained as 3D-stacks with a step-size of 2 μm by confocal microscopy using a 20 × 0.7 NA objective. Green fluorescent signals were analyzed in individual focal planes as the ratio of nuclear versus cytosolic intensities. Cells were grouped into insulin responsive (ratio < 1) versus insulin resistant (ratio ≥ 1) and the percentage of insulin resistant β-cells was calculated from the ratio of insulin resistant cells to all cells analyzed. To verify that the biosensor βIRB is expressed in pancreatic β-cells, following their transduction with the biosensor encoding adenovirus pancreatic islets were fixed and stained for insulin. As shown in Fig. 3a, cells expressing FoxO1(H215R)GFP (green) were also positive for insulin (red). Similar to insulin producing MIN6 cells, disrupting insulin signal transduction in mouse islets *in vitro* by either inhibiting Akt activity with 10 μM Akti-1/2 for 1 h (Fig. 3b) or by inhibiting insulin receptors with 100 μM HNMPA(AM)_3_ for 1h (Fig. 3c,e), but not by inhibiting signalling through the IGF1R (Fig. 3d,e), led to an increase in nuclear localization of FoxO1(H215R)GFP, i.e. insulin resistant β-cells.

In order to evoke insulin resistance by lipotoxicity as in T2DM, we incubated islets with either 0.5% BSA (control) or with 0.5 mM palmitate for up to 120 hours. 3D-stacks of images were obtained at indicated time-points after start of incubation. As shown in Fig. 3f,g, an increase in insulin resistant β-cells was observed after 72 h which increased by further incubation up to 120 h. Removal of palmitate after 120 h incubation and culturing islets further in normal fully supplemented RPMI 1640 medium for 48 h restored the cytosolic localization of the biosensor, indicating improved insulin responsiveness (Fig. 3f,g). Similar to mouse pancreatic islets, incubation of human islets with 0.5 mM palmitate for 144 h resulted in an increased nuclear localization of FoxO1(H215R)GFP (Fig. 3h–k), reflecting an increase in insulin resistant β-cells.

### *In vivo* Assessment of βIRB in Pancreatic Islets Engrafted in the ACE

**Figure 4:**
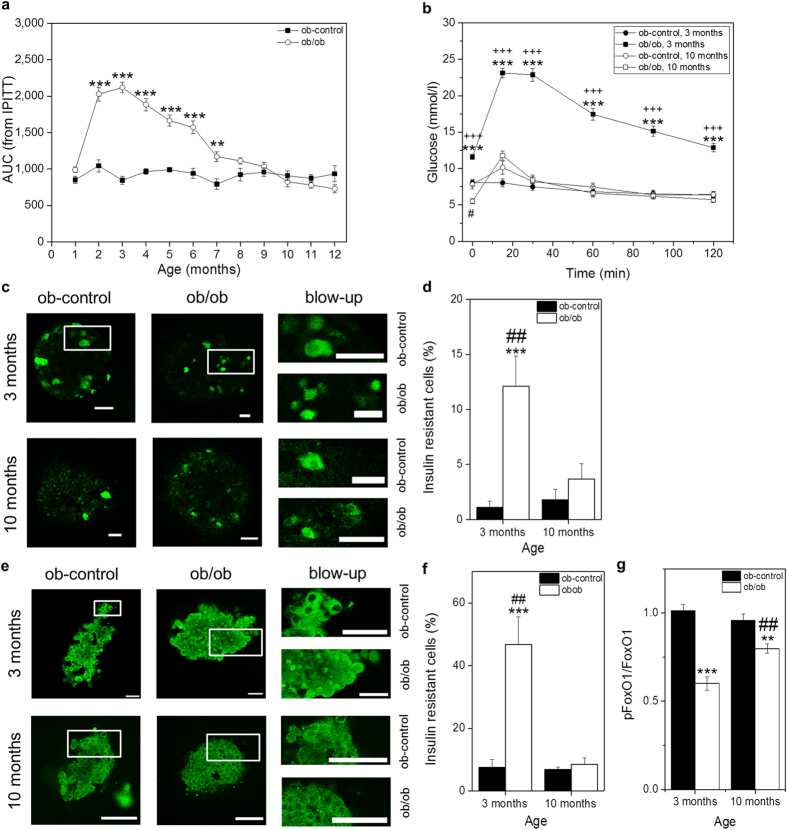
*In vivo* characterization of the insulin resistance biosensor βIRB. (**a**) Whole-body insulin resistance in ob-control and ob/ob mice from 1 to 12 months of age obtained by intraperitoneal insulin tolerance tests (IPITT^24^) and depicted as average area under the curve (AUC) of the IPITT (n = 7). **p < 0.01, ***p < 0.001. (**b**) Whole-body insulin resistance in ob-control and ob/ob mice at an age of 3 and 10 months obtained by IPITT (n = 7). ***p < 0.001: 3 months old ob-control vs. 3 months old ob/ob mice; ^+++^p < 0.001: 3 months vs. 10 months old ob/ob mice; ^#^p < 0.05: 10 months old ob-control vs. 10 months old ob/ob mice. (**c**) Representative single focal planes of engrafted islets in ob-control and ob/ob mice at 3 and 10 months of age. Scale bars, 30 μm. (**d**) *In vivo* measurement of β-cell insulin resistance in 3 and 10 months old ob/ob and ob-control mice. (n = 13) ***p < 0.001: 3 months old ob-control vs. 3 months old ob/ob mice; ^##^p < 0.01: 3 months vs. 10 months old ob/ob mice. (**e**) Representative single focal planes of FoxO-immunostained islets of 3 and 10 months old ob-control and ob/ob mice. Scale bars, 30 μm. (**f**) Subcellular distribution of endogenous FoxO1 in *in situ* islets obtained from the pancreas. (n = 4) ***p < 0.001: 3 months old ob-control vs. 3 months old ob/ob mice; ^##^p < 0.01: 3 months vs. 10 months old ob/ob. (**g**) Ratio of phosphoFoxO1 Ser256 (pFoxO1) and FoxO1 expression in 3 and 10 months old ob/ob and ob-control mice obtained by Western blot analysis. (n = 3) **p < 0.01: 10 months old ob-control vs. 10 months ob/ob mice; ***p < 0.001: 3 months old ob-control vs. 3 months ob/ob mice; ^##^p < 0.01: 3 months vs. 10 months old ob/ob mice. Data are shown as mean ± SEM. See also Supplementary Figs S2, S3 and S4.

To test βIRB *in vivo*, we compared its behaviour in control animals (C57black/B6 (B6)) and lean ob-control (ob-ctrl)) with a well established insulin resistance model, namely the ob/ob mouse. The ob/ob mouse is characterized by transient hyperinsulinemia, hyperglycemia, overall insulin resistance, islet/β-cell insulin resistance and glucose intolerance where all parameters seem to improve with increasing age^17^,^18^,^19^. Our own data show a maximum in overall insulin intolerance at three months of age which was completely restored at ten months of age (Fig. 4a,b). We isolated islets from 2-months old mice, transduced the islets *in vitro* with adenoviruses encoding βIRB and transplanted 10-20 islets into the ACE of age-matched littermate recipient mice. Islets in the ACE were first imaged four weeks after transplantation in order to allow full innervation and vascularization of the engrafted islets, i.e. at the age of three months, and continued to be imaged every month. Overall, we monitored islet insulin sensitivity in the same animal from 3 months of age up to an age of 10 months. Images were obtained as 3D-stacks with a step-size of 3 μm by confocal microscopy using a 10 × 0.3 NA objective. Green fluorescent signals were analyzed in individual focal planes as the ratio of nuclear versus cytosolic intensities. Cells were grouped into insulin responsive (ratio < 1) versus insulin resistant (ratio ≥ 1) and the percentage of insulin resistant β-cells was calculated from the ratio of insulin resistant cells to all cells analyzed. Because we observed in both control and ob/ob animals a decline in βIRB-expressing cells with time (Supplementary Fig. S1), which also occurred in transduced islets *in vitro*, we decided to re-transplant 10–20 freshly transduced age-matched islets every second month. Figure 4c,d demonstrates representative maximum projections of image stacks and respective quantification of βIRB-expressing islets in the ACE obtained in a three months old and in a ten months old ob/ob mouse as well as in ob-control mice, showing the expression and localization of FoxO1(H215R)GFP. As shown in Fig. 4d, islets in three months old ob/ob mice show a higher percentage of insulin resistant β-cells than age-matched ob-control mice. There was a decrease in nuclear localization of FoxO1(H215R)GFP in ten months old ob/ob mice, indicating an improved insulin signalling, which is in agreement with the data obtained by insulin and glucose tolerance tests from mice of the indicated age groups (Fig. 4a,b and Supplementary Fig. S2). No significant difference in nuclear localization of FoxO1(H215R)GFP, i.e. β-cell insulin sensitivity, was observed in ob-control mice and B6 mice (Supplementary Fig. S3) at three and ten months of age. In order to verify that the data obtained with the biosensor reflect the situation in *in situ* islets in the pancreas, we isolated islets from ob/ob and ob-control mice at three and ten months of age and analyzed the sub-cellular localization of endogenous FoxO1 by immunofluorescence and the phospho-FoxO1/FoxO1 ratio by Western blotting. As shown in Fig. 4e,f, in agreement with the data obtained with the biosensor in the ACE, we observed an increase in nuclear localization of endogenous FoxO1 reflecting insulin resistant β-cells in three months old ob/ob mice which was normalized in ten months old ob/ob mice. We did not observe an increase in nuclear localization of endogenous FoxO1 in ob-control mice. Western blot analysis revealed a decrease in the phospho-FoxO1/FoxO1 ratio in three months old ob/ob mice, reflecting an increased nuclear localization of FoxO1, compared to ten months old ob/ob mice, while there is no difference in this ratio in ob-control mice (Fig. 4g and Supplementary Fig. S4).

## Discussion

The aim of the present study was to develop and validate an approach allowing studies of the dynamics of insulin resistance in a specific cell type of a multicellular insulin target tissue in the living organism. As a proof-of-concept, we analyzed the dynamics of pancreatic β-cell insulin resistance in the leptin-deficient ob/ob mouse *in vivo*. The recently by us developed *in vivo* imaging platform, which provides subcellular resolution, offered the possibility to monitor insulin resistance non-invasively and longitudinally by fluorescence microscopy. For this purpose we generated a GFP-labelled biosensor based on FoxO1, a transcription factor that resides in the cytoplasm in insulin-sensitive cells and translocates into the nucleus under conditions of insulin resistance. Within the context of the micro-organ the islet of Langerhans, we achieved selective expression in pancreatic β-cells by employing the rat insulin-1 promoter to drive transcription of the biosensor in transduced islets. Besides mutating the DNA-binding site of FoxO1-GFP, thus creating FoxO1(H215R)GFP which prevented the biosensor to act as a transcription factor, all remaining parts of FoxO1 were left unchanged. Thus, the biosensor can be compared to that of endogenous FoxO1, which helped us to validate the approach by immunofluorescence and Western blot analysis of *in situ* islets. This meant that the life-cycle of the biosensor in β-cells is under the same/similar control as that of endogenous FoxO1. This might be one explanation for the decreasing expression levels of the biosensor over time due to enforced degradation. Another explanation for the decreased expression of the biosensor might be the degradation of the adenoviral transgene. Future work will have to show whether a different delivery system of the biosensor encoding transgene by for example AAV allows longer transgene survival, whether further modification of the FoxO1-moiety can stabilize the expression levels and whether increased accumulation of such a biosensor affects the physiology of the target cell. We overcame the decrease in biosensor expression by re-transplanting freshly transduced, age-matched islets into the ACE of the same animal every second month. Unfortunately, this did not allow us to monitor an individual reporter islet over the entire 8-months period, however it allowed us to monitor the same animal over the entire period and combine the *in vivo* imaging analysis of β-cell insulin resistance with overall insulin tolerance and glucose tolerance tests.

By using the βIRB reporter we may underestimate the number of insulin resistant β-cells. This is due to the fact that by using adenovirus delivered expression of the biosensor we are only able to label a limited number of cells in the periphery of the islet, while *in vitro* immunostaining includes all β-cells throughout the islet. One possibility is that the peripheral cells are affected to a lesser degree than the cells in the center of the islet. A second concern is the actual expression of the biosensor. We are only able to detect and analyze cells that express sufficiently high levels of the biosensor. In case that insulin resistance leads to a decrease in insulin promoter activity and thereby biosensor expression we are unable to monitor these cells. A solution to these problems will be the generation of a transgenic mouse by crossing RIP-CRE and a future ROSA26-CAG-Lox-Stop-Lox-βIRB mouse, which would allow CAG-promoter-driven expression of the biosensor in β-cells. In addition, we would like to emphasize that our measurement of β-cell insulin resistance, similar to *in vitro* analysis of either FoxO1 phosphorylation by Western blotting or FoxO1 localization by immunostaining, at this stage is a qualitative rather than a quantitative assessment. In this regard, the *in vivo* method demonstrates the same results as the two *in vitro* methods shown in Fig. 4c–g.

**Figure 5:**
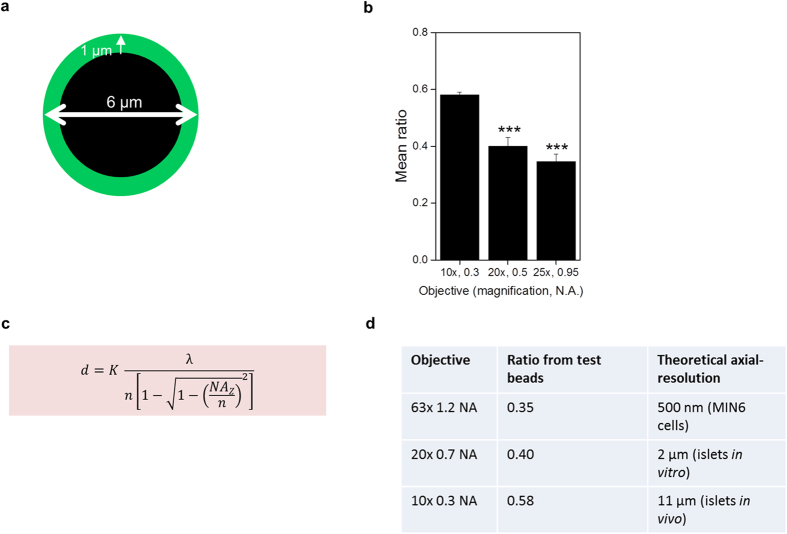
Technical and optical characteristics of the different objectives used for *in vitro* and *in vivo* imaging. (**a**) Schematic illustration of a test bead (Invitrogen, Focal Check™ fluorescence microscope test slide #1, F36909, Lot 627811) with a green fluorescent shell and a non-fluorescent core that were used to study the effect of different objectives on the core/mantle fluorescence ratio in an ideal system. (**b**) Mean ratios obtained when imaging test beads using different objectives, ***p < 0.001. (**c**) Equation for axial resolution, d, for point scan confocal microscopy where K is a scalar correction factor where for an ideal very small pinhole K_pinhole_ = 0.67, λ is the emission wavelength, n is the refractive index of medium (in our case 1.33 for water) and NA the numerical aperture was used to calculate the axial-resolution that can theoretically be reached with each objective (**d**). Data are shown as mean ± SEM, ***p < 0.001.

The data interpretation of our biosensor is based on the subcellular distribution and intensity of the GFP signal generated by FoxO1(H215R)GFP. GFP signals were analyzed in individual focal planes as the ratio of nuclear versus cytosolic intensities. Cells were grouped into insulin responsive (ratio < 1) versus insulin resistant (ratio ≥ 1) and the percentage of insulin resistant β-cells was calculated from the ratio of insulin resistant cells to all cells analyzed. In an ideal situation an insulin responsive cell would display a ratio of close to zero (no GFP in the nucleus, all GFP in the cytoplasm), however this was never observed. The ratio obtained in our experiments is dependent on the achievable Z-resolution of the objective lens used. The 63 × 1.2 NA objective, which was used when working with MIN6 cells provided a superior Z-resolution and consequently a lower limit ratio. However its size, working-distance and magnification, made it impractical to use when imaging islets either *in vitro* or *in vivo*. The 20 × 0.7 NA and 10 × 0.3 NA objectives used in those experiments reduced the Z-resolution and resulted in a higher minimal achievable ratio, but still provided enough resolution to distinguish between nucleus and cytoplasm (see Fig. 5). Despite the impact of the different objectives on the minimal achievable ratio, a cut-off of 1 reflecting a greater amount of FoxO1 in the nucleus than in the cytoplasm remained valid.

In the present study, we demonstrate that our *in vivo* imaging approach allows monitoring of pancreatic β-cell insulin sensitivity/resistance in mice over a period of eight months, i.e. from month 3 to month 10. Our data reveal that at three months of age, when ob/ob mice exhibit their maximum in overall insulin resistance, also the pancreatic β-cell is insulin resistant. Moreover, when the insulin sensitivity status of these animals improves by ten months of age, this is also reflected upon in the β-cell both in the ACE and *in situ* in the pancreas. Our approach to monitor pancreatic β-cell insulin sensitivity *in vivo* can be combined with overall insulin tolerance tests and pyruvate tolerance tests, the latter reporting on hepatic insulin sensitivity. Our technique thus for the first time allows monitoring longitudinally and non-invasively the dynamics of cell type specific insulin sensitivity/resistance in a multicellular insulin target tissue during progression and intervention of experimental T2DM.

## Methods

### Biosensor construction

pENTR2A.RIP1.FoxO1GFP was generated by replacing the cDNA for EGFP in pENTR2A.RIP1.EGFP with that of FoxO1-GFP obtained from pEGFP.N1.hFOXO1^13^. IRES-3Tomato was generated by introducing a cDNA encoding three copies of the red fluorescent protein dTomato^20^ downstream of the IRES sequence in pIRES (Clontech, Palo Alto, CA, USA). The IRES-3Tomato cassette was then introduced into pENTR2A.RIP1.FoxO1GFP thus creating pENTR2A.RIP1.FoxO1GFP-IRES-3Tomato. pENTR2A.RIP1.FoxO1(H215R)GFP-IRES-3Tomato was generated by replacing histidine (CAT) 215 by arginine (CGT) by site-directed mutagenesis by employing the QuikChange mutagenesis kit (Stratagene, LaJolla, CA, USA) and respective oligonucleotides purchased from Sigma (Paris, France). All constructions were verified by DNA sequencing. The expression cassette was transferred into the promoterless adenovirus plasmid pAd/PL-DEST (Invitrogen, Carlsbad, CA, USA) by the Gateway technique. The ViraPower Adenoviral Expression System (Invitrogen) was used to generate a replication-deficient adenovirus, which was used for transduction of cells and islets.

### Animals

The ob/ob and ob-control mice, which have a C57BL/6J background, originated from Umeå, Sweden, and were inbred in the animal core facility at Karolinska Hospital. Discrimination between ob/ob and ob-control littermates was achieved by genotypic analysis^21^. C57BL/6J (B6) mice were purchased from Charles River (Wilmington, MA, USA). All experiments were performed in accordance with the Karolinska Institutet’s guidelines for the care and use of animals in research and were approved by the institute’s Animal Ethics Committee.

### Isolation and Transduction of Pancreatic Islets

Islets were prepared from ob/ob, ob-control and B6 mice as described previously^5^. Thereafter islets were cultured in RPMI 1640 medium with a final concentration of 10% heat inactivated fetal bovine serum, 2 mM glutamine, 100 U/ml penicillin and 100 μg/ml streptomycin at 5% CO2 and 37 °C. Human islets were obtained within the Nordic Network for Islet Transplantation from deceased donors after appropriate consent. Islets were isolated at the Division of Clinical Immunology at the University of Uppsala as described previously^22^. The experiments were approved by the Regional Ethical Review Boards in Uppsala and in Stockholm. Islets were cultured in complete CMRL 1066 medium supplemented with 10% human serum. Islets were transduced with 10^7^ pfu/ml of the biosensor encoding adenovirus.

### Transplantation of Pancreatic Islets into the Anterior Chamber of the Eye (ACE)

2–3 days after transduction, islets were transplanted into the ACE of syngeneic, age-matched littermate recipients, using a technique described previously^5^. Briefly, mice were anesthetized using isoflurane. Islets were transplanted into the ACE using a glass cannula after generating a puncture in the cornea with a 27-gauge needle. Great care was taken to avoid bleeding and damage to the iris. Mice were injected s.c. with Temgesic (0.1 ml/kg; Schering-Plough, Kenilworth, NJ) for postoperative analgesia.

### *In vivo* Imaging of Intraocular Islet Grafts

Islet grafts in mice were imaged *in vivo* monthly beginning 1 month after transplantation^4^,^5^. An upright laser scanning confocal microscope (Leica TCSSP5, LEICA Microsystems, Wetzlar, Germany) equipped with a long-distance water-dipping objective (Leica HXC-APO10 × /0.30 NA) and a custom-built stereotaxic head holder allowing positioning of the mouse eye containing the engrafted islets toward the objective was used for imaging. GFP fluorescence was excited at a 488 nm and detected at 505 to 536 nm. Tomato fluorescence was excited at 561 nm and detected at 580 to 650 nm. Backscatter signal^23^ from the 561 nm excitation was collected at 555 to 565 nm. Viscotears (Novartis, Basel,Switzerland) was used as an immersion liquid between the eye and the objective, and isoflurane was used to anesthetize the mice during *in vivo* imaging. Islets were imaged as 3D-stacks with 3 μm step-size.

### Image Analysis

Images derived from either *in vivo* or *in vitro* imaging were analyzed using Leica LAS software (Leica Microsystems, Wetzlar, Germany). For each cell the central plane was determined using information from each detection channel (GFP, Tomato, backscatter). Three regions of interest were drawn manually for each cell: nucleus, cytoplasm and background, respectively (see Fig. 1). Information from all three detection channels was used to determine the position of the nucleus. To calculate the nuclear/cytoplasm-ratio, only the intensity values of the GFP channel were considered. The ratio for each cell was calculated as follows: ratio = (nucleus-background)/(cytoplasm-background). The ratios were categorized into two groups: 1) Ratios < 1 were considered nuclear FoxO1(H215R)GFP negative, since less signal is found in the nucleus than in the cytoplasm. 2) Ratios ≥ 1 were considered nuclear FoxO1(H215R)GFP positive, since more (or at least equal) signal is found in the nucleus compared in the cytoplasm. For each experimental *in vitro* or *in vivo* condition the percentage of nuclear FoxO1(H215R)GFP positive cells was calculated as follows: nuclear FoxO1(H215R)GFP (%) = cells with a ratio ≥ 1/total amount of analyzed cells. These values were considered a measure of β-cell insulin resistance and used for further statistical analysis.

### Presented Images

In the presented images green is used as digital pseudo-color for fluorescence emitted from GFP or, in the case of *in vitro* immunofluorescence images, Alexa 488; red is used as digital pseudo-color for fluorescence emitted from either Tomato or Alexa 633/insulin staining. Yellow obtained after overlaying the green and red signals indicates colocalization of the two signals. For the purpose of visibility in publication the fluorescence intensity and contrast were adjusted, while the quantification was performed on unprocessed images.

### Statistics

The values are expressed as mean ± S.E.M.. The two-sided, unpaired t-test was used to determine statistical significance between ob/ob and ob-control or B6 and the two-sided, paired t-test was used for comparison within the same strain at different ages. Statistical significance was as follows: *p < 0.05, **p < 0.01 and ***p < 0.001. IBM SPSS Statistics 22 and Microsoft office EXCEL were used for statistical analysis. *In vitro*: at least 35 FoxO1(H215R)GFP-positive fixed cells per condition were analyzed to determine the percentage of insulin resistant cells; *in vivo*: at every imaging time point 3–5 islets per animal were analyzed.

## Additional Information

**How to cite this article**: Paschen, M. *et al.* Non-invasive cell type selective *in vivo* monitoring of insulin resistance dynamics. *Sci. Rep.* **6**, 21448; doi: 10.1038/srep21448 (2016).

## Supplementary Material

Supplementary Information
