# Supplementary material for: Non-invasive cell type selective in vivo monitoring of insulin resistance dynamics
Source: Sci Rep. 2016 Feb 22;6:21448. doi: 10.1038/srep21448 (PMC4761884; doi:10.1038/srep21448)
Supplement: Supplementary Information [file srep21448-s1.pdf]

Scientific Reports
Supplementary Information to:

Non-invasive cell type selective *in vivo* monitoring of insulin resistance dynamics

Meike Paschen, Tilo Moede, Barbara Leibiger, Stefan Jacob, Galyna Bryzgalova, Ingo B. Leibiger*, Per-Olof Berggren*

The Rolf Luft Research Center for Diabetes and Endocrinology, Karolinska Institutet, SE-171 76 Stockholm, Sweden

*corresponding author

Supplementary figures

Supplementary Figure S1

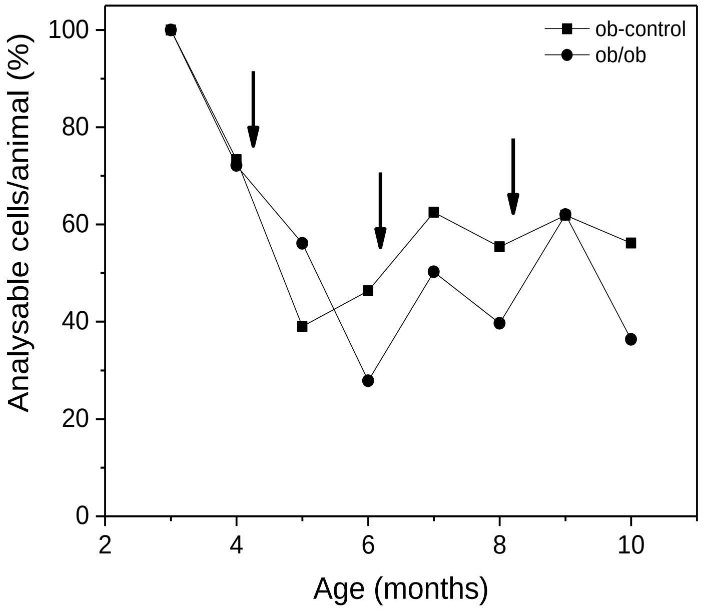

Supplementary Figure S1: Amount of cells that can be imaged and analyzed *in vivo*. Arrows indicate re-transplantation with islets of littermates (n=10 re-transplanted mice with islets of n=1-2 littermates).

Supplementary Figure S2

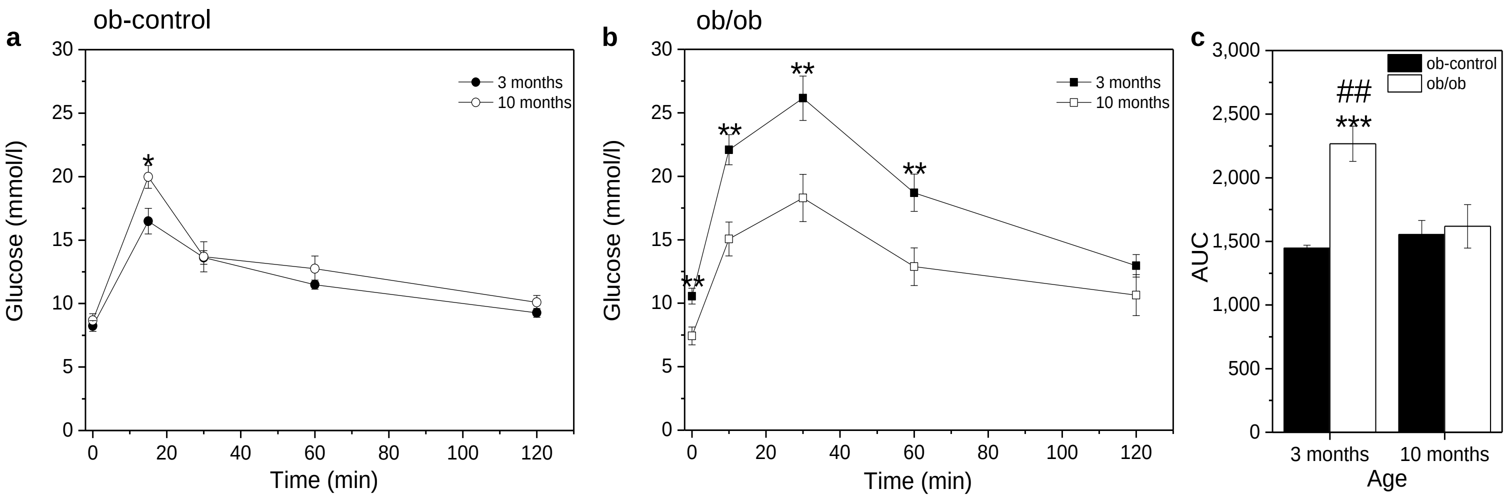

Supplementary Figure S2: IPGTT of ob-control and ob/ob mice. (Refers to Figure 4)

(a-c) IPGTT of ob-control (a) and ob/ob (b) mice at an age of 3 and 10 months (n=8) depicted as both complete traces (a, b) and area under the curve (AUC) (c), ***p < 0.001: 3 months old ob-control vs. ob/ob mice, ##p<0.01: 3 months vs. 10 months old ob/ob mice

Data are shown as mean ± SEM, *p<0.05, **p<0.01

Supplementary Figure S3

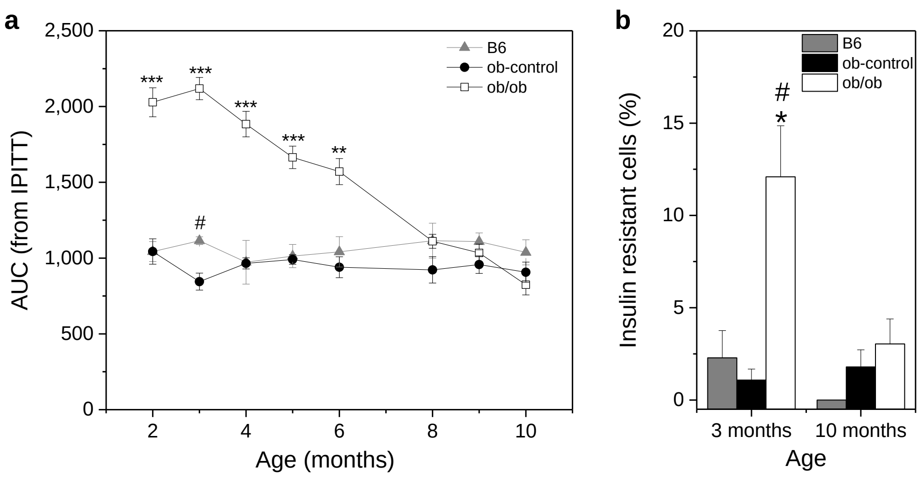

Supplementary Figure S3: Whole-body and β-cell insulin resistance in B6 mice. (Refers to Figure 4)

(a) Whole-body insulin resistance in B6, ob-control and ob/ob mice from 2 to 10 months of age obtained by IPITT and presented as average area under the curve (AUC) of the IPITT. (n=4 for B6) **$p<0.01$, ***$p<0.001$: ob/ob vs. B6; #$p<0.05$: B6 vs. ob-control. (b) *In vivo* measurement of β-cell insulin resistance in 3 and 10 months old B6, ob-control and ob/ob analyzed by using the insulin resistance biosensor. (n=3) *$p<0.05$: 3 months old ob/ob vs. B6; #$p<0.05$: 3 months vs. 10 months old ob/ob mice.

Data are shown as mean ± SEM

Supplementary Figure S4

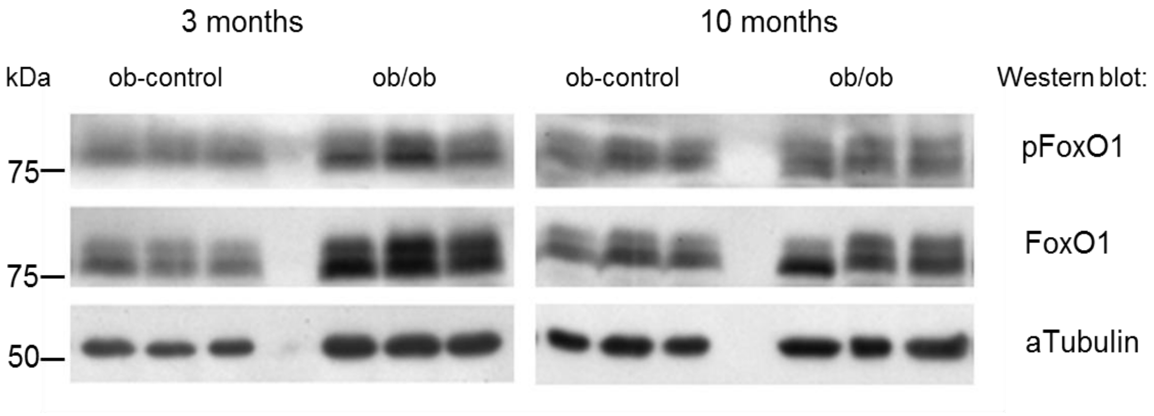

Supplementary Figure S4: FoxO1 and phospho-FoxO1 expression in ob-control and ob/ob mice. (Refers to Figure 4)

Western blot analysis of phospho-FoxO1 (pFoxO1) and total FoxO1 protein levels in 3 and 10 months old ob-control and ob/ob mice (n=3).

Supplementary methods

MIN6m9 Cells

MIN6m9 cells were cultured in DMEM medium with a final concentration of 11 mM glucose, 75 µM 2-mercaptoethanol, 10% heat inactivated fetal bovine serum and 100 U/ml penicillin and 100 µg/ml streptomycin at 5% CO_2 and 37°C. Cells were grown on 24-mm glass coverslips and incubated with 10^7 pfu/ml of the biosensor adenovirus in fully supplemented culture medium overnight. Inhibitors HNMPA(AM)$_3$ (Merck, Darmstadt, Germany), IGF-1R Inhibitor PPP (Merck), Akt kinase inhibirors Akti-1/2 or Akti-2 (Merck) were added at indicated concentrations 1 h before imaging or fixation. Since HNMPA(AM)$_3$, Akti-1/2 and Akti-2 are dissolved in DMSO, controls were incubated with equivalent amounts of DMSO.

Palmitate Treatment

Palmitate was prepared as previously described in (Cousin et al., 2001) using 5% FFA-free BSA solution to complex with palmitate (Sigma-Aldrich, St. Louis, MO, USA). 0.5% BSA solution was used as control. After transduction islets were incubated in serum free, fully supplemented RPMI 1640 medium up to 120 h with a final concentration of palmitate of 0,5 mM or 0.5% BSA. After 120 h islets were returned to fully supplemented RPMI medium.

Immunofluorescence

Freshly isolated islets were fixed with 4% paraformaldehyde in PBS for at least 48 h. They were incubated with primary antibodies anti-Foxo1 (rabbit monoclonal, 1:100, Cell Signaling, Danvers, MA) and anti-insulin (guinea pig, 1:1000, DAKO/Agilent, Santa Clara, CA, USA) in the presence of 0.1% Triton-X100 for permeabilization and 2% BSA for blocking for 48 h at room temperature. Islets were then washed three times with PBS and incubated with the secondary antibodies Alexa488-labeled anti-Rabbit and Alexa633-labeled anti-guinea pig (1:1000, Life Technologies, Carlsbad, CA, USA) in the presence of 0.1% Triton-X100 and 2%BSA. After a second wash step islets were imaged by confocal laser scanning microscopy.

Western Blotting

Islets were washed with PBS, lysed with lysis buffer (50 mM Tris, pH 7.5; 1 mM EDTA; 1 mM EGTA; 0.5 mM Na_3VO_4; 0.1% (v/v) 2-mercaptoethanol; 1% Triton X-100; 50 mM NaF; 5 nM sodium pyrophosphate; 10 mM sodium β-glycerol phosphate; 0.1 mM PMSF; 1 µg/ml of aprotinin; pepstatine leupeptin; and 1 µM Microcystin) immediately after islet isolation. The lysat was homogenized by passing it five times through an insulin syringe needle (29G) and the amount of protein was measured by the Bradford method. Equal amounts of protein were separated over a 10% SDS-polyacrylamide gel (buffering system according to Laemmli) and proteins were electrotransferred to PVDF membranes. The membranes were probed with rabbit polyclonal Phospho-FoxO1 (Ser256) antibody (Cell Signaling Technology, Denvers, MA, USA), then stripped and re-probed with rabbit monoclonal FoxO1 antibody (Cell Signaling Technology, Denvers, MA, USA) and mouse monoclonal a-Tubulin antibody (Sigma, Saint Louis, MO, USA). Immunoreactivity was detected with horseradish peroxidase-conjugated secondary antibodies using the ECL system (Amersham, Piscataway, NJ, USA).

***In vitro* Imaging of Isolated Islets and Cells**

Imaging of transduced islets and cells and immunostained islets was performed using an inverted Leica TCS-SP2 laser-scanning confocal microscope from (Leica Microsystems, Wetzlar, Germany) with the following settings: excitation wavelength 488 nm (GFP, Alexa 488), 543 nm (Tomato) and 633 nm (Alexa 633), a 488/543/633 nm triple dichroic mirror, and detection of GFP at 505 to 525 nm, Alexa 488 at 505 to 535 nm, Tomato at 605 to 670 nm and Alexa 633 at 640 to 680 nm. For MIN6m9 cells single images at a central plane of each cell were obtained using the Leica HCX PL APO 63x/1.20 NA objective lens. Isolated islets were imaged as 3D stacks with a 2µm step size utilizing the Leica PL APO CS 20x/0.7 NA objective lens.

Physiological Measurements

At least seven mice were used for body weight measurement, intraperitoneal (i.p.) glucose tolerance test (IPGTT) and insulin tolerance tests (IPITT). IPGTT and IPITT were carried out in 6 h fasted mice.

Intraperitoneal Glucose Tolerance Test (IPGTT)

To determine glucose tolerance, blood glucose levels were measured in mice fasted for 6 h at basal state (0 min) and 10, 30, 60 and 120 min after glucose injection (2 g/kg body weight, dissolved in PBS, i.p.). Glucose concentrations were determined using the Accu-Chek Aviva monitoring system (Roche, Basel, Switzerland).

Intraperitoneal Insulin Tolerance Test (IPITT)

In IPITTs, the rate of glucose disappearance from blood after an insulin challenge was determined and was performed as described earlier in (Ref. 24, Gao et al., Mol Endocrinol 20, 1287-99, 2006). Blood glucose concentration was measured in mice fasted for 6 h at basal state (0 min). Then, mice were injected with insulin (0.25 U/kg body weight, diluted in PBS, i.p.) followed by glucose administration (1 g/kg body weight, i.p.) and blood glucose concentrations were determined at 15, 30, 60, 90 and 120 min after glucose injection. Combining the administration of insulin with glucose in our IPITT reduces the risk of severe hypoglycemia in older ob/ob mice, which have low blood glucose levels.
